# When AI threatens my future career: growth mindset and perceived employability in Chinese college students' career decision making anxiety

**DOI:** 10.3389/fpsyg.2026.1933842

**Published:** 2026-09-22

**Authors:** Huizhi Chen

**Affiliations:** School of General Education, Wenzhou Polytechnic University, Wenzhou, China

**Keywords:** AI employment threat perception, career decision-making anxiety, college students, growth mindset, moderated mediation model, perceived employability

## Abstract

**Background:**

Artificial intelligence (AI) is rapidly reshaping the labor market and may intensify college students' uncertainty and anxiety about future career decisions. This study examined the psychological mechanism linking AI employment threat perception to career decision-making anxiety, focusing on the mediating role of perceived employability and the moderating role of growth mindset.

**Methods:**

A questionnaire survey was conducted among 526 college students in China. Hierarchical regression, bootstrap mediation analysis, moderation analysis, simple slope analysis, and moderated mediation analysis were used to test the proposed model.

**Results:**

AI employment threat perception positively predicted career decision-making anxiety and negatively predicted perceived employability. Perceived employability negatively predicted career decision-making anxiety and partially mediated the relationship between AI employment threat perception and career decision-making anxiety. Growth mindset significantly moderated the relationship between AI employment threat perception and perceived employability, such that the negative effect was weaker among students with a higher growth mindset. The moderated mediation analysis further showed that growth mindset weakened the indirect effect of AI employment threat perception on career decision-making anxiety through perceived employability.

**Conclusions:**

AI employment threat may increase college students' career decision-making anxiety both directly and indirectly by reducing perceived employability. Growth mindset serves as an important psychological resource that buffers this negative process. These findings provide implications for career education, employability development, and psychological support in higher education.

## 1 Introduction

The rapid development of artificial intelligence (AI) is reshaping occupational tasks and skill demands in the labor market and is increasingly influencing how college students perceive employment risks and evaluate their future careers (Septiandri et al., 2024; Liang and Zhai, 2025; Duan et al., 2026). As generative AI, algorithmic systems, and automated technologies become increasingly embedded in recruitment, work processes, and performance evaluation, students must reconsider whether their professional knowledge and skills will remain valuable in an AI-driven work environment. AI and automation may substitute for some occupational tasks while simultaneously changing the types of human capabilities demanded by employers (Acemoglu and Restrepo, 2020; Frey and Osborne, 2017; Septiandri et al., 2024). For students who have not yet fully entered the labor market, such changes may generate uncertainty about employment opportunities, skill relevance, and future career paths. Recent studies have accordingly linked AI-related perceptions with career decision making, employment risk, unemployment anxiety, and career anxiety among university students (Duan et al., 2026; Liang and Zhai, 2025; Uçar et al., 2025; Dag et al., 2026; Yaşar and Karagucuk, 2025).

Career decision-making anxiety is particularly relevant in this context. It refers to the tension, worry, hesitation, and uncertainty individuals experience when choosing career goals, evaluating employment directions, and planning future career development (Arbona et al., 2021; Li et al., 2026). Career decisions are inherently uncertain because individuals must integrate information about their interests, abilities, educational backgrounds, and labor market opportunities (Gati et al., 1996; Savickas, 2005; Wang and Li, 2024), and anxiety has been associated with career indecision and reduced career certainty (Campagna and Curtis, 2007; Germeijs et al., 2006; Li et al., 2026). AI-related labor market change may intensify this uncertainty when students believe that technological development threatens their future employment prospects.

In this study, AI employment threat perception refers to students' subjective judgment that AI may replace future jobs, increase employment thresholds, reduce the value of their professional knowledge, or destabilize future career paths. Unlike general employment pressure arising from job availability or labor market competition, this perception specifically concerns technological substitution, skill devaluation, and AI-driven changes in career opportunities (Wang et al., 2025; Liu et al., 2026). The study draws primarily on cognitive appraisal theory, which proposes that psychological responses to environmental change depend on how individuals evaluate both potential threats and their capacity to cope with them (Lazarus and Folkman, 1984; Zhang et al., 2026). Within this framework, AI employment threat perception represents an appraisal of potential career threat, whereas perceived employability reflects students' evaluation of whether they possess sufficient career resources to respond to that threat. Perceived employability concerns individuals' subjective assessment of their capacity to obtain employment, meet job requirements, and adapt to labor market change (Rothwell et al., 2008; Vanhercke et al., 2014; Wut et al., 2025; Ho et al., 2026).

This interpretation is also consistent with conservation of resources theory and career construction theory, which emphasize the importance of valued resources and adaptive capacity under changing environmental conditions (Hobfoll, 1989; Hobfoll et al., 2018; Savickas, 2005; Wang and Li, 2024; Yang et al., 2025). In an AI-driven labor market, students may increasingly evaluate their employability in terms of technological adaptability, AI-related skills, and continuous learning capacity (Wut et al., 2025; Ramos et al., 2025). Growth mindset, defined as the belief that abilities and skills can be developed through effort, learning, and strategic adjustment (Dweck, 2006; Yeager and Dweck, 2020; Liu et al., 2026), may further shape this appraisal process by influencing whether students regard their career resources as fixed or developable. Accordingly, this study aims to examine how AI employment threat perception is associated with career decision-making anxiety, whether perceived employability explains this relationship, and whether growth mindset weakens the negative association between AI employment threat perception and perceived employability.

Students who perceive stronger AI employment threat are likely to experience greater career decision-making anxiety because anticipated job substitution, skill devaluation, and changing employment requirements can make future career choices appear less predictable and controllable. Cognitive appraisal theory suggests that anxiety becomes more likely when environmental changes are evaluated as threatening and difficult to manage (Lazarus and Folkman, 1984; Zhang et al., 2026). Consistent with this reasoning, AI-related anxiety and impact awareness have been associated with unemployment anxiety, employment risk perception, and career-related concerns among university students (Uçar et al., 2025; Dag et al., 2026; Duan et al., 2026; Liang and Zhai, 2025). Therefore:

H1: AI employment threat perception is positively associated with career decision-making anxiety among college students.

AI employment threat perception may also weaken students' perceived employability. When students believe that AI may reduce the long-term value of their professional knowledge or substantially raise future skill requirements, they may question whether their existing abilities remain sufficient for the labor market. Such perceptions can signal potential loss or insufficiency of career resources (Hobfoll, 1989; Antonio and Chiesa, 2024). This concern is increasingly relevant as AI literacy, digital adaptability, and AI-related competencies become part of students' assessments of employment competitiveness (Wut et al., 2025; Ramos et al., 2025). Related research has also connected AI-related concerns with perceived future employability and broader career orientations (Tuncal and Çeken, 2025; Jiang and Chen, 2026). Therefore:

H2: AI employment threat perception is negatively associated with perceived employability among college students.

Perceived employability should, in turn, be negatively associated with career decision-making anxiety. Students who believe that they possess sufficient skills, employment opportunities, and adaptive capacity are more likely to view career choices as manageable rather than uncontrollable. This reasoning is consistent with social cognitive career theory, which emphasizes self-efficacy and outcome expectations in career-related choices (Lent et al., 1994; Li et al., 2025; Liu et al., 2026). Previous research has similarly linked perceived employability with more positive career adaptation and lower career distress and decision-making difficulties (Li et al., 2023; Li and He, 2025; Shi et al., 2025; Xiong et al., 2025). Consequently:

H3: Perceived employability is negatively associated with career decision-making anxiety among college students.

These relationships further suggest an indirect process. AI-related technological change may become psychologically consequential when students interpret it not only as an external threat but also as evidence that their own career resources are insufficient. Perceived employability therefore connects environmental threat appraisal with students' confidence in their capacity to adapt to future employment conditions. Research has similarly identified employability and related psychological resources as important for career adaptation under uncertainty (Ho et al., 2026; Lee et al., 2025; Kwak et al., 2025; Yang et al., 2025). Thus:

H4: Perceived employability mediates the relationship between AI employment threat perception and career decision-making anxiety among college students.

Growth mindset may weaken this process because students who view abilities as developable are more likely to believe that emerging skill gaps can be addressed through learning and adaptation. When facing AI-related employment change, these students may be more willing to acquire AI skills, develop digital competencies, and adjust their career preparation rather than interpret technological change as permanent evidence of declining professional value. Growth mindset has also been associated with career decision-making self-efficacy and career adaptability (Li et al., 2025; Puspitasari, 2024; Liu et al., 2026). Therefore:

H5: Growth mindset moderates the relationship between AI employment threat perception and perceived employability among college students, such that the negative association is weaker among students with a higher growth mindset.

Taken together, the proposed model integrates AI employment threat as a threat appraisal, perceived employability as an appraisal of career resources, and growth mindset as a personal resource shaping this appraisal process. By doing so, the study seeks to explain not only whether perceived AI-related employment threat is associated with greater career decision-making anxiety, but also how this association may emerge and why its strength may vary across students. The proposed model is presented in Figure 1.

**Figure 1:**
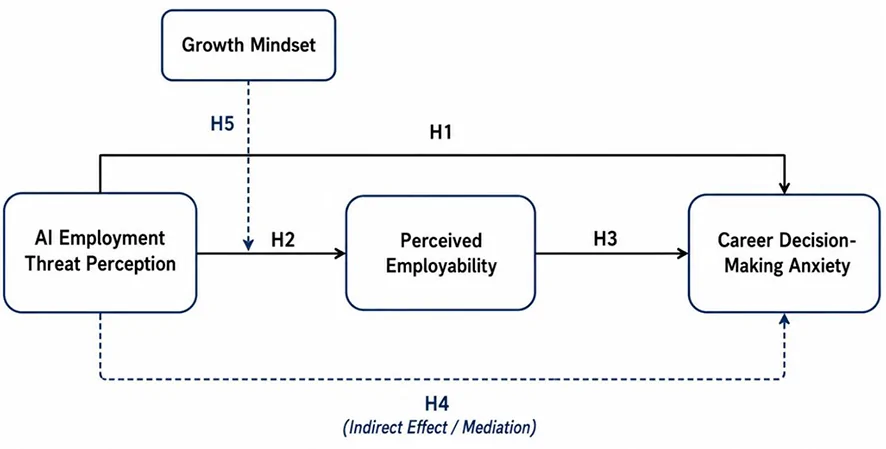
Research model. Source: Created by the authors.

The research model is shown in Figure 1:

## 2 Methods

### 2.1 Participants and data collection

This study adopted a questionnaire survey design. Participants were college students from a university in Zhejiang Province, China. The sample included students from different grades, major categories, and levels of AI tool use experience to improve sample diversity. All methods were performed in accordance with relevant guidelines and regulations, as well as the ethical principles of the Declaration of Helsinki. The study protocol and all survey procedures were reviewed and approved by the Ethics Committee of the School of General Education, Wenzhou Polytechnic.

Before completing the questionnaire, all participants read an informed consent statement. They were informed that the study was conducted only for academic purposes, that their responses would be anonymous, and that participation was voluntary. Informed consent was obtained from all participants before data collection. As all participants were 18 years of age or older, legal guardian consent was not required.

A total of 600 questionnaires were distributed. To ensure data quality, responses with excessively short completion time, obvious patterned answers, failed attention checks, or substantial missing values on key variables were removed. Finally, 526 valid responses were retained, with an effective response rate of 87.67%. Among the valid participants, 314 were female, accounting for 59.7%, and 212 were male, accounting for 40.3%. Participants ranged in age from 18 to 24 years, with a mean age of 20.32 years and a standard deviation of 1.31.

### 2.2 Measures

All core variables were measured using established scales or items adapted from prior research and revised to fit the context of AI employment and college students' career development. Except for demographic variables, all items were measured on a seven point Likert scale ranging from 1 = strongly disagree to 7 = strongly agree.

AI employment threat perception measured students' subjective judgment that AI may replace jobs, reduce the value of their major, raise employment thresholds, and increase career uncertainty. The items were adapted from research on AI anxiety, AI impact awareness, and employment risk perception (Duan et al., 2026; Liang and Zhai, 2025; Uçar et al., 2025). Perceived employability measured students' confidence in obtaining employment opportunities, meeting job requirements, and coping with labor market competition. The items were adapted from the self perceived employability scale for university students (Rothwell et al., 2008; Wut et al., 2025). Career decision making anxiety measured students' tension, worry, and uncertainty when making career choices, judging employment directions, and planning future career development. The items were adapted from research on career decision making difficulties, career anxiety, and career choice anxiety (Campagna and Curtis, 2007; Gati et al., 1996; Li et al., 2026). Growth mindset measured the extent to which students believed that abilities and skills can be developed through effort, learning, and strategic adjustment. The items were adapted from growth mindset research (Dweck, 2006; Yeager and Dweck, 2020; Li et al., 2025; Liu et al., 2026). For scales originally developed in English, a translation and back translation procedure was used. A small scale pretest was conducted before the formal survey to ensure that the items were clear and appropriate for college students.

### 2.3 Control variables

This study controlled for gender, age, grade, major category, AI tool use frequency, and whether students had taken a career planning course. Gender and age may be related to employment pressure perception and career development stage. Grade is associated with the proximity of career decision making tasks. Major category may influence perceived AI substitution risk. AI tool use frequency may affect students' familiarity with AI and their perception of AI threat. Career planning course experience may influence career decision making confidence. Therefore, these variables were included as controls in subsequent model testing.

### 2.4 Measurement model assessment

Before hypothesis testing, the reliability, convergent validity, and discriminant validity of the measurement model were examined. Internal consistency was assessed using Cronbach's alpha (Cronbach, 1951; Cheung et al., 2024) and composite reliability (Fornell and Larcker, 1981; Cheung et al., 2024), while convergent validity was evaluated using average variance extracted (AVE; Fornell and Larcker, 1981; Cheung et al., 2024). Confirmatory factor analysis (CFA) was used to evaluate the measurement structure and model fit (Jöreskog, 1969; Kline, 2023). The Cronbach's alpha values of all constructs were above 0.90, indicating good internal consistency. Composite reliability values were above 0.90, average variance extracted values were above 0.70, and standardized factor loadings were above.80. These results indicate satisfactory convergent validity. The detailed results are shown in Table 1.

**Table 1:** Reliability and convergent validity.

Construct	Items	Mean	SD	Standardized loading range	Cronbach's α	CR	AVE
AI employment threat perception	5	4.256	1.074	0.877–0.889	0.929	0.947	0.780
Perceived employability	5	4.548	1.010	0.888–0.902	0.936	0.952	0.797
Career decision making anxiety	5	4.113	1.053	0.882–0.892	0.932	0.949	0.787
Growth mindset	4	4.821	0.937	0.887–0.906	0.917	0.942	0.802

A confirmatory factor analysis was then conducted to examine the four factor measurement model. The results showed good model fit, χ^2^/df = 1.13, CFI = 0.988, TLI = 0.987, RMSEA = 0.016, and SRMR = 0.026. Compared with the three factor, two factor, and single factor models, the four factor model showed clearly better fit, indicating that AI employment threat perception, perceived employability, career decision making anxiety, and growth mindset were empirically distinct. The model comparison results are shown in Table 2.

**Table 2:** Measurement model comparison.

Model	χ^2^	df	χ^2^/df	CFI	TLI	RMSEA	SRMR
Four factor model	165.57	146	1.13	0.988	0.987	0.016	0.026
Three factor model: perceived employability and career decision making anxiety combined	1235.74	149	8.29	0.867	0.848	0.118	0.074
Two factor model: AI employment threat perception and other variables combined	2558.40	151	16.94	0.706	0.667	0.174	0.141
Single factor model	4082.21	152	26.86	0.520	0.460	0.222	0.183

Discriminant validity was assessed using the Fornell–Larcker criterion (Fornell and Larcker, 1981; Cheung et al., 2024), together with the heterotrait–monotrait ratio (HTMT) as a complementary assessment (Henseler et al., 2015; Cheung et al., 2024). As shown in Table 3, the square root of AVE for each construct was higher than its correlations with other constructs, indicating satisfactory discriminant validity.

**Table 3:** Discriminant validity.

Variable	1	2	3	4
1. AI employment threat perception	0.883			
2. Perceived employability	−0.390	0.893		
3. Career decision making anxiety	0.477	−0.628	0.887	
4. Growth mindset	−0.087	0.359	−0.236	0.895

Diagonal values are the square roots of AVE. Off diagonal values are inter construct correlations.

HTMT values were also calculated. All HTMT values were below 0.85, with the highest value being 0.672, further supporting discriminant validity.

### 2.5 Common method bias

Because this study used self reported data collected at a single time point, common method bias may be a concern (Podsakoff et al., 2024). Several procedural remedies were adopted. First, the questionnaire was completed anonymously to reduce social desirability bias. Second, participants were informed that there were no right or wrong answers and were asked to respond according to their actual perceptions. Third, items measuring different constructs were presented in a mixed order to reduce consistency based responding. Finally, attention check items were included, and invalid responses were removed during data screening.

A statistical check was also conducted using confirmatory factor analysis. As shown in Table 2, the single factor model showed poor fit and was substantially worse than the theoretically expected four factor model. This result suggests that common method bias was unlikely to seriously threaten the validity of the findings.

### 2.6 Analytical strategy

The analyses were conducted in several stages. First, the measurement properties of the four focal constructs were assessed as described in Section 2.4. Second, descriptive statistics and bivariate correlations were examined. Third, hierarchical regression analyses were conducted to test the direct relationships among AI employment threat perception, perceived employability, and career decision-making anxiety after controlling for gender, age, grade, major category, AI tool use frequency, and career planning course experience. Fourth, the mediating effect of perceived employability was examined using 5,000 bootstrap resamples and 95% confidence intervals (Hayes, 2022). For the moderation analysis, AI employment threat perception and growth mindset were mean-centered before constructing their interaction term. Significant interactions were further examined using simple slope analyses at one standard deviation below and above the mean of growth mindset. Finally, conditional indirect effects and the index of moderated mediation were estimated using 5,000 bootstrap resamples. An effect was considered statistically significant when its 95% bootstrap confidence interval did not include zero.

## 3 Results

### 3.1 Descriptive statistics and correlation analysis

Descriptive statistics and correlation analyses were first conducted for the main variables. As shown in Table 4, AI employment threat perception was positively correlated with career decision making anxiety, indicating that students who perceived stronger AI related employment threat tended to report higher levels of career decision making anxiety. AI employment threat perception was negatively correlated with perceived employability, suggesting that stronger AI employment threat perception was associated with lower evaluation of one's employability. Perceived employability was negatively correlated with career decision making anxiety, indicating that students with stronger perceived employability tended to experience lower anxiety in career choice and future planning. Growth mindset was positively correlated with perceived employability, while its correlations with AI employment threat perception and career decision making anxiety were not significant. Overall, the correlation results were consistent with the expected directions.

**Table 4:** Descriptive statistics and correlations.

Variable	M	SD	1	2	3	4
1. AI employment threat perception	4.28	0.88	1			
2. Perceived employability	4.58	0.89	−0.130^**^	1		
3. Career decision making anxiety	4.11	0.92	0.225^***^	−0.313^***^	1	
4. Growth mindset	4.83	0.89	−0.084	0.172^***^	−0.034	1

*N* = 526.

^**^p < 0.01.

^***^p < 0.001.

### 3.2 Hypothesis testing

Hierarchical regression and bootstrap analyses were used to test the hypotheses. The results are shown in Table 5.

**Table 5:** Results of direct, mediation, and moderation effects.

Test type	Path/effect	b/effect	SE/boot SE	*t*	*p*	95% CI
Direct effect	AI employment threat perception → Career decision-making anxiety	0.240	0.044	5.385	< 0.001	–
Direct effect	AI employment threat perception → Perceived employability	−0.137	0.043	−3.169	0.002	–
Direct effect	Perceived employability → Career decision-making anxiety	−0.303	0.043	−7.007	< 0.001	–
Mediation effect	AI employment threat perception → Perceived employability → Career decision-making anxiety	0.042	0.015	–	–	[0.013, 0.073]
Direct effect after mediator	AI employment threat perception → Career decision-making anxiety	0.198	0.043	4.608	< 0.001	[0.113, 0.284]
Moderation effect	AI employment threat perception × Growth mindset → Perceived employability	0.122	0.049	2.473	0.014	–
Simple slope analysis						
Growth mindset level		b	SE	t	*p*	
Low growth mindset (-1 SD)		−0.229	0.060	−3.795	< 0.001	
High growth mindset (+1 SD)		−0.013	0.062	−0.213	0.832	

*N* = 526. Control variables include gender, age, grade, major category, AI tool use frequency, and career planning course experience. Mediation effects were tested using 5,000 bootstrap samples. CI, confidence interval.

After controlling for gender, age, grade, major category, AI tool use frequency, and career planning course experience, AI employment threat perception significantly and positively predicted career decision making anxiety, *b* = 0.240, SE = 0.044, *t* = 5.385, *p* < 0.001. This result indicates that students with stronger AI employment threat perception experienced higher career decision making anxiety, supporting H1.

AI employment threat perception also significantly and negatively predicted perceived employability, *b* = −0.137, SE = 0.043, *t* = −3.169, *p* = 0.002. This result suggests that students who perceived stronger AI employment threat tended to report lower perceived employability, supporting H2. In addition, perceived employability significantly and negatively predicted career decision making anxiety, b = −0.303, SE = 0.043, *t* = −7.007, *p* < 0.001, indicating that students with higher perceived employability experienced lower career decision making anxiety. Thus, H3 was supported.

To examine the mediating role of perceived employability, a bootstrap analysis with 5,000 resamples was conducted. The indirect effect of AI employment threat perception on career decision making anxiety through perceived employability was significant, Effect = 0.042, Boot SE = 0.015, 95% CI [0.013, 0.073]. Because the confidence interval did not include zero, H4 was supported. After perceived employability was included in the model, the direct effect of AI employment threat perception on career decision making anxiety remained significant, Effect = 0.198, SE = 0.043, *t* = 4.608, *p* < 0.001, 95% CI [0.113,0.284], indicating a partial mediation effect.

To test the moderating role of growth mindset, AI employment threat perception and growth mindset were mean centered, and their interaction term was created. The interaction between AI employment threat perception and growth mindset significantly and positively predicted perceived employability, b = 0.122, SE = 0.049, *t* = 2.473, *p* = 0.014. This result indicates that growth mindset buffered the negative effect of AI employment threat perception on perceived employability, supporting H5.

A simple slope analysis was further conducted. Under low growth mindset, AI employment threat perception significantly and negatively predicted perceived employability, b = −0.229, SE = 0.060, *t* = −3.795, *p* < 0.001. Under high growth mindset, the effect of AI employment threat perception on perceived employability was not significant, *b* = −0.013, SE = 0.062, *t* = −0.213, *p* = 0.832. These results suggest that students with a stronger growth mindset were less likely to reduce their perceived employability when facing AI employment threat.

### 3.3 Moderated mediating effect

The moderated mediation effect was further examined using 5,000 bootstrap samples. As shown in Table 6, the indirect effect of AI employment threat perception on career decision-making anxiety through perceived employability was significant under low growth mindset, Effect = 0.069, Boot SE = 0.023, 95% CI [0.027, 0.119]. However, this indirect effect became nonsignificant under high growth mindset, Effect = 0.004, Boot SE = 0.019, 95% CI [−0.033, 0.042]. The difference between the two conditional indirect effects was significant, Effect = −0.065, Boot SE = 0.026, 95% CI [−0.119, −0.018]. The index of moderated mediation was also significant, Index = −0.037, Boot SE = 0.017, 95% CI [−0.075,−0.008], indicating that growth mindset weakened the indirect effect of AI employment threat perception on career decision-making anxiety through perceived employability.

**Table 6:** Results of the moderated mediation effect.

Growth mindset level	Indirect effect	Boot SE	95% CI
Low growth mindset	0.069	0.023	[0.027, 0.119]
High growth mindset	0.004	0.019	[−0.033, 0.042]
Difference	−0.065	0.026	[−0.119, −0.018]
Index of moderated mediation	−0.037	0.017	[−0.075, −0.008]

## 4 Discussion

### 4.1 Main findings and theoretical contributions

This study examined how AI employment threat perception influences college students' career decision making anxiety by developing and testing a moderated mediation model. The results showed that AI employment threat perception significantly increased career decision making anxiety. In other words, when students more strongly perceived that AI may replace future jobs, reduce the value of their major, or raise employment thresholds, they were more likely to experience anxiety, uncertainty, and hesitation in career choice, employment direction judgment, and future career planning. This finding suggests that the influence of AI on college students is not limited to changing skill demands but is also associated with how students psychologically evaluate their future careers (Liang and Zhai, 2025; Uçar et al., 2025; Duan et al., 2026). By focusing specifically on career decision-making anxiety, the present study shows that perceived AI-related employment threat is closely associated with students' uncertainty and hesitation when evaluating future career options.

The results also showed that perceived employability partially mediated the relationship between AI employment threat perception and career decision making anxiety. AI employment threat perception not only directly increased career decision making anxiety, but also intensified anxiety by weakening students' evaluation of their own employability. This means that AI threat does not simply make students feel that the employment environment is more difficult. It also changes how students evaluate their abilities, professional value, and future competitiveness. When students believe that their abilities are insufficient to meet the requirements of the AI era, they are more likely to experience helplessness and uncertainty in career decision making. Perceived employability has been regarded as an important psychological resource for students before entering the labor market (Vanhercke et al., 2014; Ho et al., 2026). This study further shows that, in the context of AI employment threat, perceived employability is not only a positive resource for career development, but also a key mechanism explaining how technological threat is translated into career anxiety.

In addition, growth mindset significantly moderated the relationship between AI employment threat perception and perceived employability. The simple slope analysis showed that AI employment threat perception significantly reduced perceived employability among students with a low growth mindset, whereas this negative effect became nonsignificant among students with a high growth mindset. The moderated mediation analysis further showed that growth mindset weakened the indirect effect of AI employment threat perception on career decision making anxiety through perceived employability. This suggests that growth mindset does not eliminate students' perception of AI employment risk, but it changes how students interpret that risk. Students who believe that abilities can be developed through learning and effort are more likely to view AI driven job changes as opportunities for skill renewal and career adaptation, rather than as a direct denial of their employment value. This finding is consistent with the view that growth mindset helps individuals respond to challenges, maintain learning motivation, and strengthen career decision confidence (Yeager and Dweck, 2020; Li et al., 2025).

Recent studies of Chinese college students provide a useful basis for comparing and interpreting these findings. Duan et al. (2026) showed that AI anxiety was associated with poorer career decision outcomes among Chinese college students, with career adaptability serving as an important explanatory mechanism. Liang and Zhai (2025) similarly found that awareness of AI-related change was associated with stronger employment risk perception, partly through employment anxiety, while individual learning orientation altered the strength of this process. These studies and the present findings converge in suggesting that students' responses to AI-related labor market change depend not only on perceptions of technological threat but also on the psychological and career resources through which such threat is interpreted. The present study extends this emerging evidence by identifying perceived employability as a career-specific resource appraisal linking AI employment threat perception with career decision-making anxiety. In addition, recent studies of Chinese university students have shown that growth mindset is positively associated with career decision-making self-efficacy and related psychological resources (Li et al., 2025; Kong and Zhang, 2026; Liu et al., 2026). Whereas these studies primarily demonstrate the positive role of growth mindset in developing career resources, the present results further indicate a buffering role: the negative association between AI employment threat perception and perceived employability was substantially weaker among students with a stronger growth mindset. Taken together, these comparisons suggest that AI-related career responses among Chinese college students are shaped by the interaction between perceived technological threat and the personal resources available to interpret and adapt to such change.

This study makes three theoretical contributions. First, it develops a more integrated explanation of how AI-related labor market change becomes psychologically relevant to college students' career decision making. Drawing primarily on cognitive appraisal theory, the findings suggest that AI employment threat can be understood as a career-related threat appraisal that is associated with students' subsequent evaluations of their capacity to cope with technological change. This perspective extends research on career decision-making anxiety by showing how an emerging technological condition enters the appraisal process underlying career uncertainty. It also places AI employment threat within a broader process of evaluating environmental demands and personal career resources, rather than treating it simply as another source of general employment pressure.

Second, the study identifies perceived employability as an important resource-appraisal mechanism linking AI employment threat perception with career decision-making anxiety. The findings show that students' responses to AI-related career threats are associated with how they evaluate their own capacity to remain competitive and adaptable in the future labor market. Perceived employability therefore provides a theoretical bridge between an externally perceived technological threat and an internally experienced career response. By positioning perceived employability within the appraisal process, the study also complements career construction and conservation of resources perspectives by highlighting the subjective evaluation of career resources as an important component of students' adaptation to technological change.

Third, the findings clarify an important boundary condition of this appraisal process by identifying growth mindset as a personal resource that shapes students' responses to AI employment threat. The negative relationship between AI employment threat perception and perceived employability was substantially weaker among students with a stronger growth mindset, and the corresponding indirect association with career decision-making anxiety was also reduced. This result suggests that the psychological consequences of technological threat depend partly on whether students view their abilities and skills as developable under changing labor market conditions. Integrating growth mindset into the appraisal framework therefore helps explain heterogeneity in students' responses to similar AI-related career threats and extends growth mindset research into the emerging context of AI-driven career change.

### 4.2 Practical implications

The findings provide several practical implications for career education and employment support in higher education. First, universities should help students develop a more accurate appraisal of AI-related career change. Career education can provide occupation- and major-specific information about how AI is changing particular tasks, skill requirements, and career pathways. Providing concrete information about which tasks are more exposed to automation and which human capabilities remain important may help students evaluate AI-related employment change more realistically and reduce anxiety arising from vague or exaggerated perceptions of technological substitution.

Second, interventions should place greater emphasis on strengthening students' perceived employability. The mediating results indicate that students' evaluation of their own career resources is closely associated with their anxiety when facing AI-related employment threats. Universities can therefore combine career counseling with concrete employability-building activities, including AI literacy training, digital skill development, interdisciplinary projects, internships, and opportunities to apply AI tools in profession-specific tasks. Career services can also help students identify transferable skills and recognize how existing competencies can be adapted to emerging occupations. Such interventions may strengthen both actual career resources and students' confidence in their capacity to respond to labor market change.

Third, growth-oriented career support may be particularly useful for students who experience stronger AI employment threat. The moderation results suggest that students with a stronger growth mindset are less likely to associate perceived AI threat with reduced employability. Career counselors and instructors can therefore use growth-oriented feedback, examples of successful skill transitions, structured reflection on learning progress, and individualized development plans to reinforce the idea that career-relevant abilities can be continuously developed. This approach may be especially valuable when students interpret technological change as evidence that their existing professional training has permanently lost value.

Finally, universities may benefit from adopting more differentiated career support strategies. Students reporting simultaneously high AI employment threat, low perceived employability, and a weaker growth mindset may represent a particularly vulnerable group. Screening these perceptions during career counseling could help identify students who require more intensive support. For these students, combining accurate labor market information, concrete skill-development opportunities, and psychological support may be more effective than providing general reassurance about the future of AI and employment.

### 4.3 Limitations and future research directions

This study has several limitations. First, the cross-sectional survey design limits causal and temporal inference. Because mediation processes are inherently temporal, relationships estimated from cross-sectional data may not accurately represent how these processes unfold over time (Maxwell and Cole, 2007; Georgeson et al., 2025). Although the proposed moderated mediation model is theoretically grounded, the temporal ordering among AI employment threat perception, perceived employability, and career decision-making anxiety cannot be established. Career decision-making anxiety may also influence how strongly students perceive AI-related employment risks, and reciprocal relationships remain possible. Future research could use multi-wave longitudinal, cross-lagged, or experimental designs to provide stronger evidence regarding temporal precedence and causal direction.

Second, all focal variables were assessed using self-reported perceptions from the same respondents. Such designs may be affected by shared method variance and response tendencies, concerns that remain relevant in contemporary behavioral research (Podsakoff et al., 2003, 2024). Although the procedural remedies and measurement-model comparisons used in this study reduce concerns about severe common method bias, they cannot eliminate it completely. Moreover, AI employment threat perception reflects students' subjective appraisal of technological risk rather than objective occupational exposure to AI. Future studies could therefore combine self-reported psychological measures with external or behavioral indicators, such as occupation- or major-specific AI exposure, AI-use behavior, skill-development activities, internship performance, or job-search outcomes.

Third, the sample was drawn from students within a single institutional context in China, which limits the extent to which the findings can be generalized to other populations and settings. The broader problem of generalizing psychological findings beyond the specific samples and contexts from which they are obtained has received sustained methodological attention (Henrich et al., 2010; Yarkoni, 2022). Although participants represented different grades and major categories, institutional, regional, cultural, and disciplinary differences may shape both perceived AI employment threat and career decision-making processes. Replication across universities, regions, countries, educational levels, and academic disciplines would therefore help establish the robustness and boundary conditions of the proposed model.

Finally, the proposed model captures only part of the broader process through which students respond to AI-related career change. The present study focuses on perceived employability and growth mindset, while other resources—including AI literacy, career adaptability, digital self-efficacy, social support, and future time perspective—may also shape students' responses. In addition, the model emphasizes threat-oriented appraisal, although appraisal theory recognizes that environmental change may be interpreted as either threatening or potentially manageable, and recent AI research similarly suggests that individuals can perceive AI in terms of both threat and opportunity (Lazarus and Folkman, 1984; Wang et al., 2025; Zhang et al., 2026). Future research could therefore examine a broader set of personal and contextual resources and simultaneously consider AI-related threat and opportunity appraisals.

### 4.4 Conclusion

The aim of this study was to examine how AI employment threat perception is associated with college students' career decision-making anxiety, with particular attention to the mediating role of perceived employability and the moderating role of growth mindset. Overall, the results supported all five hypotheses. AI employment threat perception was positively associated with career decision-making anxiety (H1) and negatively associated with perceived employability (H2), while perceived employability was negatively associated with career decision-making anxiety (H3). Perceived employability partially mediated the association between AI employment threat perception and career decision-making anxiety, supporting H4. Growth mindset moderated the relationship between AI employment threat perception and perceived employability, such that the negative association was weaker among students with a higher growth mindset, supporting H5. The moderated mediation analysis further showed that the indirect association between AI employment threat perception and career decision-making anxiety through perceived employability was weaker at higher levels of growth mindset. Taken together, these findings support the proposed moderated mediation framework and highlight the roles of perceived employability and growth mindset in understanding college students' responses to AI-related career uncertainty.

## Data Availability

The raw data supporting the conclusions of this article will be made available by the authors, without undue reservation.
