# Supplementary material for: When AI threatens my future career: growth mindset and perceived employability in Chinese college students' career decision making anxiety
Source: Front Psychol. 2026 Sep 22;17:1933842. doi: 10.3389/fpsyg.2026.1933842 (PMC13638579; doi:10.3389/fpsyg.2026.1933842)
Supplement: Supplementary file 1 [file Supplementary_file_1.docx]

**Appendix A. Measurement Items**

All items were measured on a seven point Likert scale ranging from 1 = strongly disagree to 7 = strongly agree.

| **Construct** | **Code** | **Measurement item** |
| --- | --- | --- |
| AI employment threat perception | AETP1 | I am concerned that the development of artificial intelligence will reduce my future job opportunities. |
|  | AETP2 | I worry that artificial intelligence may weaken the employment value of my major. |
|  | AETP3 | I believe that artificial intelligence will make it more difficult for me to find a suitable job in the future. |
|  | AETP4 | I am concerned that artificial intelligence will reduce my future career competitiveness. |
|  | AETP5 | The development of artificial intelligence makes me feel uncertain about my future employment prospects. |
| Perceived employability | PE1 | I believe that I have the ability to obtain a suitable job after graduation. |
|  | PE2 | I believe that my professional knowledge and skills can meet future job requirements. |
|  | PE3 | Even in a competitive labor market, I am confident that I can find an appropriate job. |
|  | PE4 | I believe that I have the ability to adapt to future changes in the labor market. |
|  | PE5 | Overall, I am confident about my future employment prospects. |
| Career decision making anxiety | CDMA1 | I feel anxious when thinking about my future career choices. |
|  | CDMA2 | I worry that I may not be able to make the right career decision. |
|  | CDMA3 | I feel uncertain when deciding on my future employment direction. |
|  | CDMA4 | I am afraid that I may choose a career path that is not suitable for me. |
|  | CDMA5 | Career planning often makes me feel stressed. |
| Growth mindset | GM1 | People can improve their abilities through effort and learning. |
|  | GM2 | I can develop new career skills if I use appropriate learning strategies. |
|  | GM3 | When facing technological changes, I believe that I can gradually adapt. |
|  | GM4 | Career related abilities can be improved through continuous learning. |
